# Supplementary material for: OncoPan®: An NGS-Based Screening Methodology to Identify Molecular Markers for Therapy and Risk Assessment in Pancreatic Ductal Adenocarcinoma
Source: Biomedicines. 2022 May 23;10(5):1208. doi: 10.3390/biomedicines10051208 (PMC9138989; doi:10.3390/biomedicines10051208)
Supplement: Supplementary file 1 [file biomedicines-10-01208-s001.zip › biomedicines-1723870-supplementary.pdf]

biomedicines

MDPI

Supplementary Materials

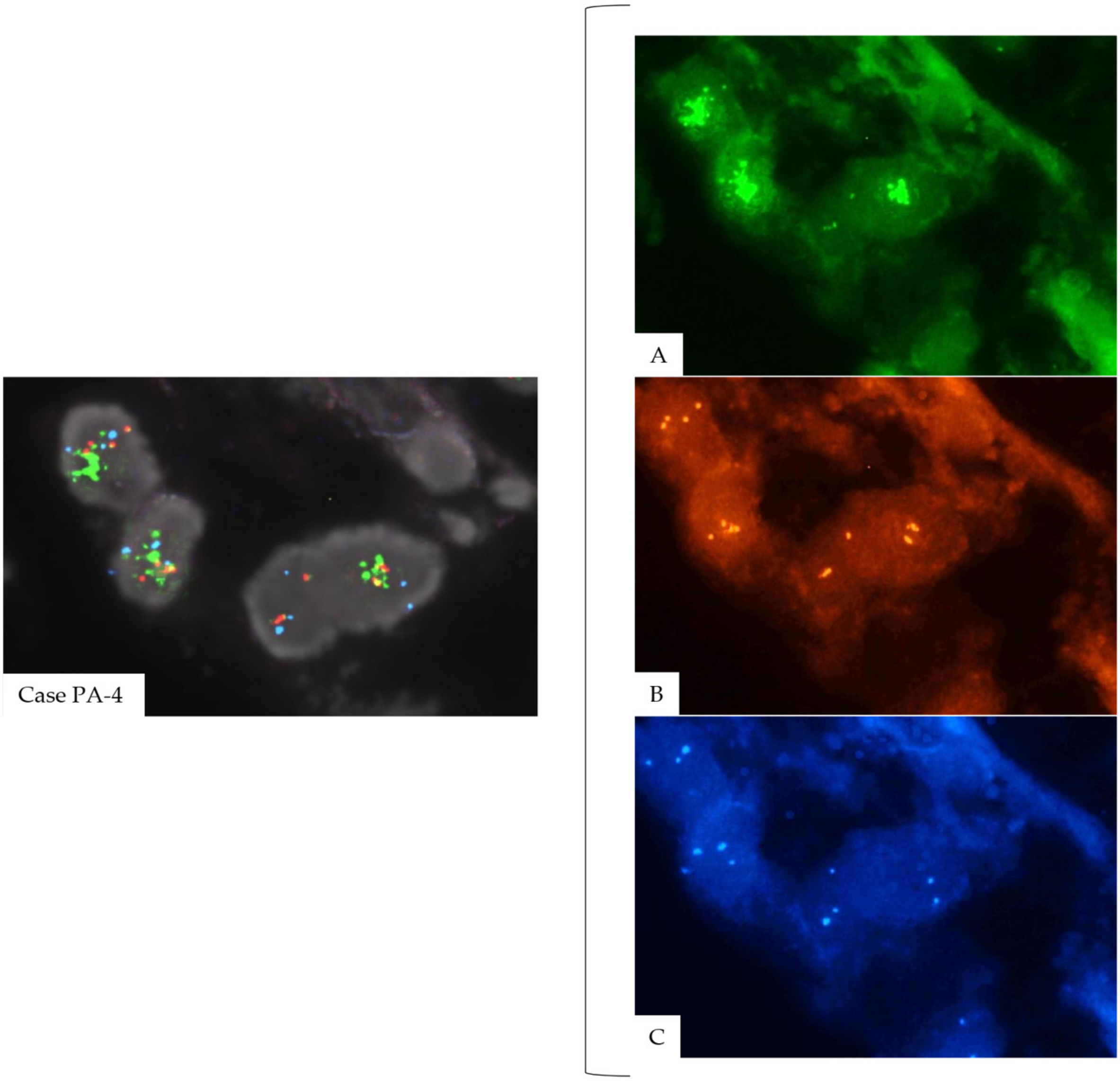

Figure S1

Figure S1. FISH analysis on PA-4 sample using Zytolight SPEC *HER2*/*TOP2A*/CEN17 Triple Color Probe (Zytovision). Imagies were obtained with Bioview system (Abbott) and fluorescence microscope at 1200× magnification: (**A**) merge of *HER2* signals (green), *TOP2A* signals (red) and chromosome 17 centromere signals (aqua). FISH analysis revealed amplification of *HER2* gene, diploidy and polisomy of *TOP2A* region and diploidy and polisomy of chromosome 17.

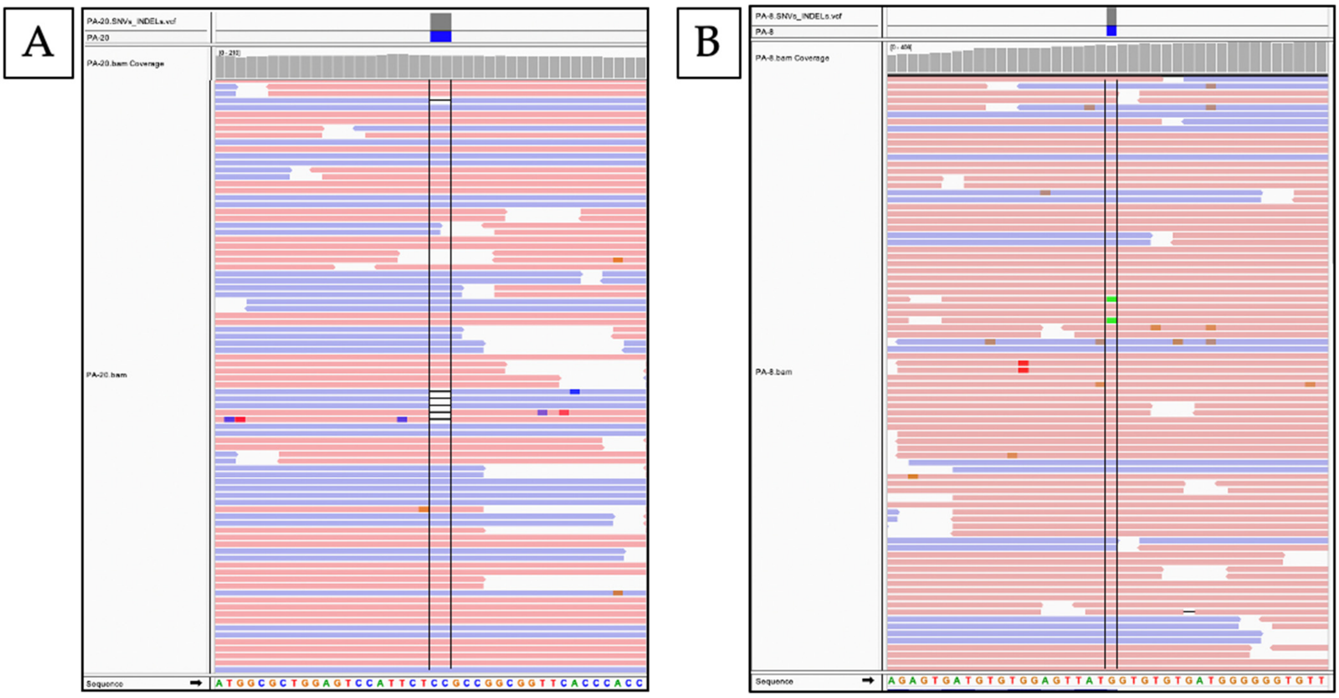

Figure S2. The picture shows the Integrative Genomics Viewer (IGV) analysis of the *HER2* variants: A) c.2685_2686del p.(Arg896Profs*8) in PA20 sample and B) c.2725G>A p.(Gly909Ser) in PA8 sample. Each track comprises three parts: a bicolor histogram that identifies the variant position (upper pane); a grey histogram of the read depth (middle pane); the reads as aligned to the reference sequence (lower pane).

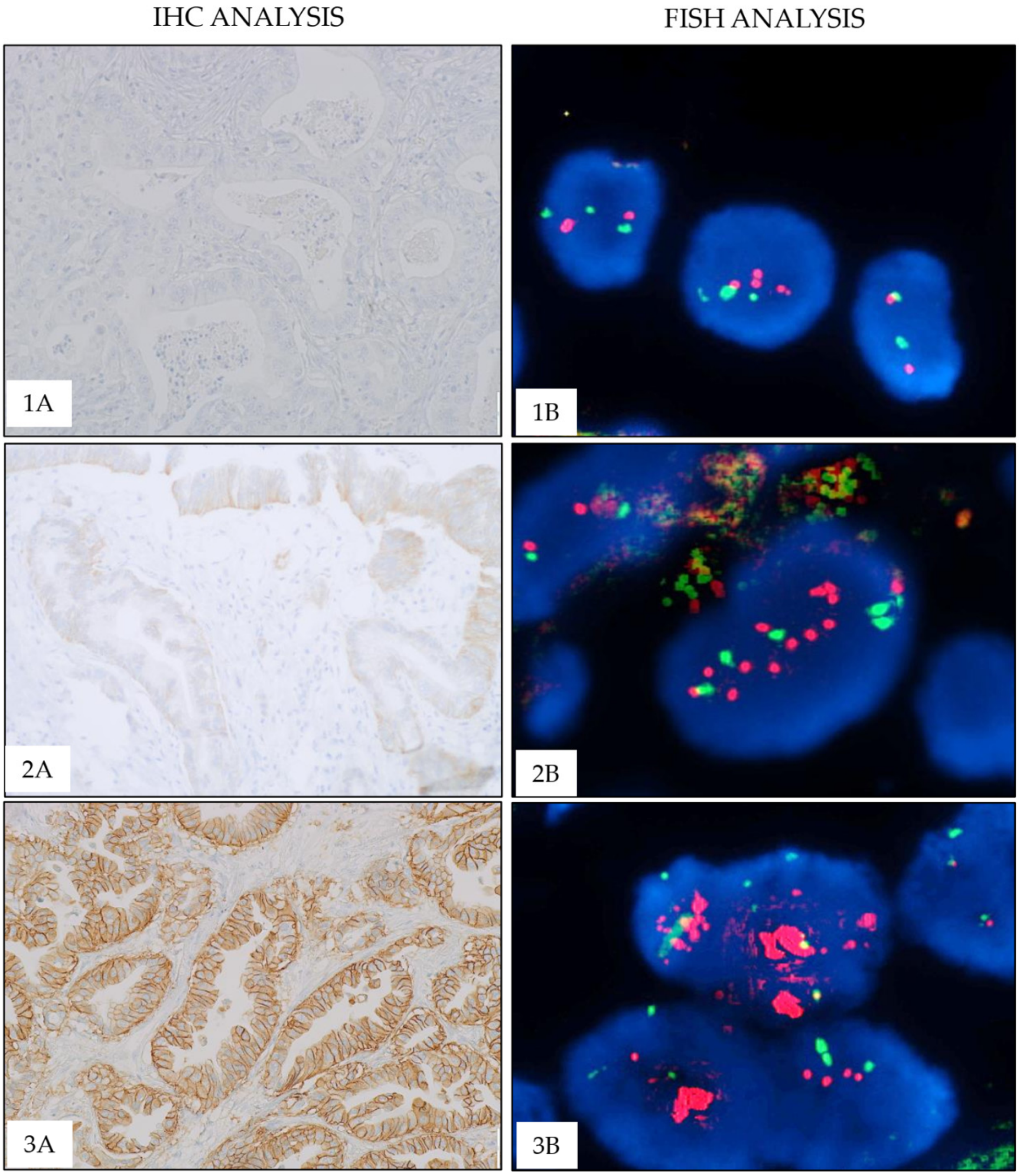

Figure S3. Immunohystochemical and FISH results: case PA3 showing negative *HER2* expression (1A) and absence of *HER2* gene amplification (1B); case PA1 showing weak/moderate *HER2* expression (2A) and *HER2* amplification (2B); case PA4 showing *HER2* overexpression (3A) and gene amplification (3B, red signal). Immunohistochemistry, DAB-hematoxylin; FISH imagines were obtained using and fluorescence microscope at 1200× magnification and Bioview system (Abbott).

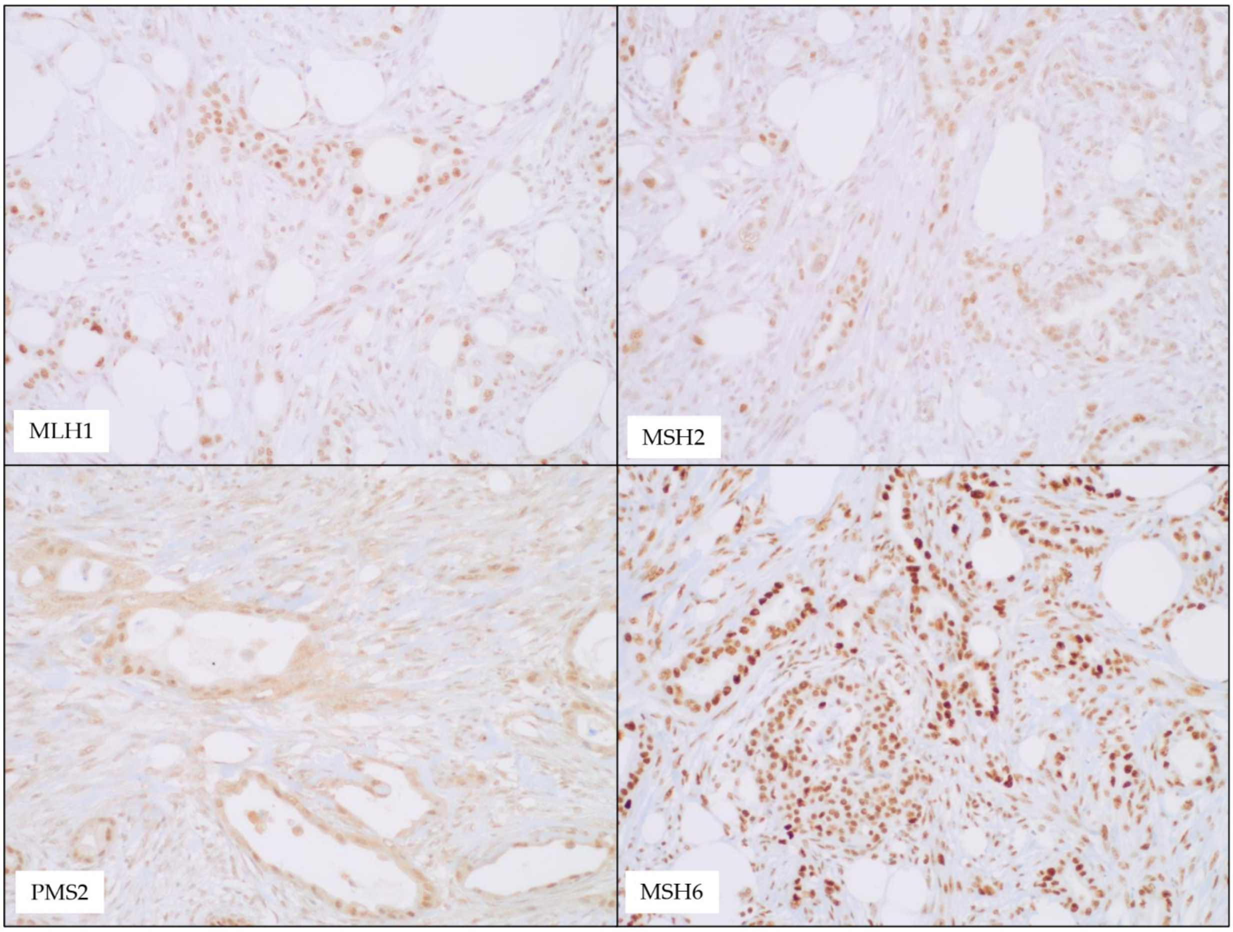

Figure S4. Immunohystochemical normal expression of MMR proteins in PA12.

Table S1. List of 37 genes and 2 pseudogenes included in the Oncopan® panel _v2.

**Gene**	**GenBank accession number**	**Locus Genomic Reference**	**Associated syndrome**	**Risk level**
APC	NM_000038	LRG_130t1	*APC*-associated polyposis<br>Pancreatic cancer	High<br>Low
ATM	NM_000051	LRG_135t1	Ataxia-telangectasia<br>Hereditary breast/pancreatic/prostate cancer	Biallelic mutations: high<br>Heterozygotes: moderate at most
BARD1	NM_000465	LGR_297t1	Hereditary breast cancer	Low / moderate
BMPR1A	NM_004329	LRG_298t1	Juvenile polyposis	High
BRAF	NM_004333	LRG_299t1	NO	NO
BRCA1	NM_007294	LRG_292t1	Hereditary breast/ovarian cancer<br>Pancreatic cancer	High<br>Moderate
BRCA2	NM_000059	LRG_293t1	Hereditary breast/ovarian cancer<br>Pancreatic cancer	High<br>Moderate
BRIP1	NM_032043	LRG_300t1	Ovarian Cancer	Low / moderate
CDH1	NM_004360	LRG_301t1	Diffuse hereditary gastric cancer	High
CDKN2A (p16INK4a)<br>CDKN2A (p.14ARF)	NM_000077<br>NM_058195	LRG_11t1<br>LRG_11t2	Hereditary melanoma<br>Pancreatic cancer	High<br>Moderate / High
CDK4	NM_000075	LRG_490t1	Hereditary melanoma	High
CHEK2	NM_007194	LRG_302t1	Hereditary breast cancer	Moderate
CHEK2P2	NR_038836	CHEK2 pseudogene	NO	NO
CTNNA1	NM_001903	NO	Diffuse hereditary gastric cancer	Uncertain – presumed high from limited case reports

EGFR	NM_005228	LRG_304t1	NO	NO
EPCAM	NM_002354	LRG_215t1	Lynch syndrome	High
HER2	NM_004448	LRG_724t2	NO	NO
FANCM	NM_020937	LRG_502t1	Hereditary breast cancer	Low / Moderate
KRAS	NM_033360	LRG_344t2	NO	NO
MLH1	NM_000249	LRG_216t1	Lynch syndrome<br>Pancreatic cancer	High<br>Moderate
MSH2	NM_000251	LRG_218t1	Lynch syndrome<br>Pancreatic cancer	High<br>Moderate
MSH6	NM_000179	LRG_219t1	Lynch syndrome<br>Pancreatic cancer	High<br>Moderate
MSH3	NM_002439	NO	MSH3- associated Polyposis	Biallelic mutations: Uncertain – presumed high from limited case reports
MUTYH	NM_001128425	LRG_220t1	*MUTYH*-associated polyposis	Biallelic mutations: high<br>Heterozygotes: uncertain – moderate at most
NBN	NM_002485	LRG_158t1	Hereditary breast cancer	Low
NRAS	NM_002524	LRG_92t1	NO	NO
NTHL1	NM_002528	LRG_1366t1	Recessive adenomatous polyposis	Uncertain – presumed high from limited case reports
PALB2	NM_024675	LRG_308t1	Hereditary breast cancer<br>Pancreatic cancer	Moderate-high<br>Moderate
PIK3CA	NM_006218	LRG_310t1	NO	NO
PMS2	NM_000535	LRG_161t1	Lynch syndrome	Moderate-High
PMS2CL	NR_002217	PMS2 pseudogene	NO	NO

POLD1	NM_002691	LRG_785t1	Polymerase proofreading-associated polyposis (PPAP)	Uncertain – presumed high from limited case reports
POLE	NM_006231	LRG_789t1	Polymerase proofreading-associated polyposis (PPAP)	Uncertain – presumed high from limited case reports
PTEN	NM_000314	LRG_311t1	Cowden syndrome	Moderate-high
RAD51C	NM_058216	LRG_314t1	Hereditary ovarian cancer	Moderate
RAD51D	NM_002878	LRG_516t1	Hereditary ovarian cancer	Moderate
SMAD4	NM_005359	LRG_318t1	Juvenile polyposis	High
STK11	NM_000455	LRG_319t1	Peutz-Jeghers syndrome<br>Pancreatic cancer	High<br>High
TP53	NM_00546	LRG_321t1	Li Fraumeni syndrome	High

Table S2. - List of variants detected by the Oncopan® panel_v2. Only pathogenic (class5), probably pathogenic (class4) and variants of uncertain significance (class3) are included.

Sample ID	Gene	Mutation type	Nucleotide HGVS[a]	Protein HGVS[a]	dbSNP [b]	AF%[c]	Classification
PA1	*KRAS*	Missense	c.35G>T	p.(Gly12Val)	rs121913529	20%	P
	MSH3	Missense	c.1088C>A	p.(Thr363Asn)	NA	11%	VUS
	SMAD4	Nonsense	c.247C>T	p.(Gln83*)	NA	12%	P
	TP53	Missense	c.817C>T	p.(Arg273Cys)	rs121913343	26%	P
PA2	*KRAS*	Missense	c.34G>C	p.(Gly12Arg)	rs121913530	45%	P
	TP53	Missense	c.380C>T	p.(Ser127Phe)	rs730881999	56%	P
PA3	*CDKN2A*	Frameshift	c.45_88del	p.(Trp15Cysfs*14)	NA	17%	P
	KRAS	Missense	c.34G>C	p.(Gly12Arg)	rs121913530	17%	P
	BRCA1	Large deletion	c.(4986+1_4987-1)_(5074+1_5075-1)del	p.(Val1665Serfs*8)	NA	NQ	P
	TP53	Frameshift	c.216dup	p.(Val73Argfs*16)	rs730882018	21%	P
PA4	*KRAS*	3'UTR	c.*4592G>A	3'UTR	rs536562107	24%	VUS
	PIK3CA	Missense	c.2499G>A	p.(Met833Ile)	NA	19%	VUS
	SMAD4	Frameshift	c.1237_1249del	p.(Tyr413Lysfs*19)	NA	22%	P
	TP53	Missense	c.377A>G	p.(Tyr126Cys)	NA	29%	P
PA5	*KRAS*	Missense	c.35G>A	p.(Gly12Asp)	rs121913529	8%	P
	TP53	Nonsense	c.880G>T	p.(Glu294*)	rs1057520607	14%	P
PA6	*CDKN2A*	Missense	c.423C>A	p.(Asn141Lys)	NA	42%	VUS
	KRAS	Missense	c.35G>A	p.(Gly12Asp)	rs121913529	37%	P
	TP53	Nonsense	c.772G>T	p.(Glu258*)	NA	38%	P
PA7	*KRAS*	Missense	c.35G>A	p.(Gly12Asp)	rs121913529	28%	P
	PMS2/PMS2CL [d]	Missense	*c.2380C>T*	p.(Pro794Ser)	rs773393960	15%	VUS

PA8	*BRCA1*	Missense	c.2910A>C	p.(Lys970Asn)	rs431825394	37%	VUS
	CHEK2	Missense	c.1116_1117delinsTG	p.(Lys373Glu)	NA	22%	VUS
	HER2	Missense	c.2725G>A	p.Gly909Ser	NA	2%	VUS
	KRAS	Missense	c.34G>C	p.(Gly12Arg)	rs121913530	9%	P
	SMAD4	Nonsense	c.1324C>T	p.(Gln442*)<br>NA		26%	P
	TP53	Frameshift	c.455dup	p.(Pro153Alafs*28)	rs730882019	22%	P
PA9	*ATM*	Missense	c.1846A>G	p.(Thr616Ala)	rs587780615	29%	VUS
	BRCA2	Missense	c.8771A>C	p.(Glu2924Ala)	NA	25%	VUS
	BRCA2	Missense	c.8968T>G	p.(Trp2990Gly)	NA	29%	VUS
	KRAS	Missense	c.35G>A	p.(Gly12Asp)	rs121913529	3%	P
	MSH2	Nonsense	c.301G>T	p.(Glu101*)	rs63750318	25%	P
	MSH2	Missense	c.2703A>C	p.(Glu901Asp)	NA	20%	VUS
	PIK3CA	Missense	c.1082A>G	p.(Tyr361Cys)	NA	44%	VUS
	PIK3CA	Missense	c.2T>A	p.(?)	NA	20%	VUS
	PIK3CA	Missense	c.2119G>A	p.(Glu707Lys)	rs3729687	40%	VUS
PA10	*CHEK2*	Intronic	c.320-5T>A	-	rs121908700	51%	VUS
	KRAS	Missense	c.35G>A	p.(Gly12Asp)	rs121913529	5%	P
	POLE	Nonsense	c.5687C>A	p.(Ser1896*)	NA	9%	VUS
	PTEN	Nonsense	c.871G>T	p.(Glu291*)	NA	4%	VUS
	RAD51D	Missense	c.698A>G	p.(Glu233Gly)	rs28363284	51%	B
	TP53	Nonsense	c.298C>T	p.(Gln100*)	NA	17%	P
PA11	*FANCM*	Missense	c.5584G>A	p.(Val1862Met)	rs1280753248	44%	VUS
	POLD1	Missense	c.3016G>A	p.(Ala1006Thr)	rs376197467	42%	VUS
	CDKN2A	Frameshift	c.131dup	p.(Tyr44*)	rs730881673	5%	P

PA12	*KRAS*	Missense	c.35G>T	p.(Gly12Val)	rs121913529	3%	P
	MSH6	Frameshift	c.3126_3172+38del	p.?	NA	2%	LP
PA13	*KRAS*	Missense	c.35G>T	p.(Gly12Val)	rs121913529	16%	P
	MSH2	Missense	c.435T>G	p.(Ile145Met)	rs63750124	40%	VUS
	PMS2/PMS2CL [d]	Missense	c.1148A>T	p.(Asn383Ile)	NA	12%	VUS
PA14	*PALB2*	Missense	c.2353C>T	p.(Pro785Ser)	rs730881889	45%	VUS
	PIK3CA	Missense	c.1930T>C	p.(Tyr644His)	rs17849072	15%	VUS
PA15	*BRCA1*	Intronic	c.5074+107C>T	-	rs373676607	16%	VUS
	BRCA2	Frameshift	c.2585_2586insC	p.(Lys862Asnfs*19)	NA	12%	P
	KRAS	Missense	c.35G>A	p.(Gly12Asp)	rs121913529	22%	P
	TP53	Missense	c.818G>A	p.(Arg273His)	rs28934576	41%	P
PA16	*BRIP1*	Missense	c.550G>T	p.(Asp184Tyr)	rs201047375	31%	VUS
	CTNNA1	Missense	c.2671G>A	p.(Val891Met)	rs771903880	52%	VUS
	TP53	Nonsense	c.437G>A	p.(Trp146*)	rs1206165503	5%	P
PA17	*BRCA2*	Missense	c.8480C>T	p.(Pro2827Leu)	NA	6%	VUS
	CDH1	Missense	c.1489G>A	p.(Glu497Lys)	NA	9%	VUS
	CDKN2A	Missense	c.242C>A	p.(Pro81His)	NA	37%	VUS
	KRAS	Missense	c.35G>A	p.(Gly12Asp)	rs121913529	10%	P
	SMAD4	Missense	c.736C>A	p.(Pro246Thr)	rs876659967	52%	VUS
	TP53	Missense	c.743G>A	p.(Arg248Gln)	rs11540652	22%	P
PA18	*BRCA1*	Missense	c.2395A>T	p.(Asn799Tyr)	NA	4%	VUS
	CHEK2	Missense	c.1392G>T	p.(Lys464Asn)	rs764396738	6%	VUS
	KRAS	Missense	c.35G>A	p.(Gly12Asp)	rs121913529	11%	P
	MSH2	In frame deletion	c.4_78del	p.Ala2_Met26del	NA	4%	VUS

	MSH6	In frame deletion	c.1957_2010del	p.Val653_Gly670del	NA	10%	VUS
	PMS2	Missense	c.1004A>G	p.(Asn335Ser)	rs200513014	44%	VUS
	SMAD4	Missense	c.353C>T	p.(ala118Val)	NA	6%	P
PA19	*MSH6*	Missense	c.866G>C	p.(Gly289Ala)	rs368318845	47%	VUS
PA20	*CHEK2*	Missense	c.1116_1117delinsTG	p.(Lys373Glu)	NA	14%	VUS
	HER2	Frameshift	c.2685_2686del	p.(Arg896Profs*8)	NA	4%	LP
	FANCM	Nonsense	c.4270C>T	p.(Arg1424*)	rs751954386	20%	P
	KRAS	Missense	c.183A>C	p.(Gln61His)	rs17851045	16%	P
	POLE	Missense	c.5036G>A	p.(Arg1679His)	rs748940418	9%	VUS
PA21	*CDKN2A*	Frameshift	c.131dup	p.(Tyr44*)	rs730881673	14%	P
	KRAS	Missense	c.34G>C	p.(Gly12Arg)	rs121913530	13%	P
	MUTYH	Missense	c.251A>G	p.(Tyr84Cys)	rs200747973	50%	VUS
	TP53	Missense	c.844C>T	p.(Arg282Trp)	rs28934574	20%	P

Abbreviations: NA, not available; NQ, not quantifiable; P, pathogenic; LP, likely pathogenic; VUS, variant of uncertain significance; B, benign. [a] Mutation nomenclature according to the *Human Genome Variation Society* (HGVS http://varnomen.hgvs.org). [b] Variant reference according to the *Single Nucleotide Polymorphism Database (dbSNP http://www.ncbi.nlm.nih.gov/SNP)* [c] Mutant Allelic Frequency [d] Variant in either PMS2 or PMS2CL. Nomenclature referred to PMS2. Variants investigated by Sanger sequencing on DNA extracted from non-neoplastic tissue.
